# Empirical insights into the stochasticity of small RNA sequencing

**DOI:** 10.1038/srep24061

**Published:** 2016-04-07

**Authors:** Li-Xuan Qin, Thomas Tuschl, Samuel Singer

**Affiliations:** 1 Department of Epidemiology and Biostatistics, Memorial Sloan Kettering Cancer Center, New York, USA; 2 Laboratory of RNA Molecular Biology, The Rockefeller University, New York, USA; 3 Department of Surgery, Memorial Sloan Kettering Cancer Center, New York, USA.

## Abstract

The choice of stochasticity distribution for modeling the noise distribution is a fundamental assumption for the analysis of sequencing data and consequently is critical for the accurate assessment of biological heterogeneity and differential expression. The stochasticity of RNA sequencing has been assumed to follow Poisson distributions. We collected microRNA sequencing data and observed that its stochasticity is better approximated by gamma distributions, likely because of the stochastic nature of exponential PCR amplification. We validated our findings with two independent datasets, one for microRNA sequencing and another for RNA sequencing. Motivated by the gamma distributed stochasticity, we provided a simple method for the analysis of RNA sequencing data and showed its superiority to three existing methods for differential expression analysis using three data examples of technical replicate data and biological replicate data.

Next-generation sequencing is a stochastic, or “noisy”, process^1^. An intrinsic source of the noise is the inherent randomness of the biochemical processes for library preparation and read generation^2^. Thus, repeated sequencing of the same sample (i.e., “technical replication”) can result in different sequencing reads^3^. A proper understanding of the noise distribution is critical for choosing the right distributional model to make accurate statistical inference, and consequently for the accurate assessment of biological heterogeneity and of differential expression for individual genes.

In the literature the intrinsic stochasticity for RNA sequencing has been assumed to follow a Poisson distribution. For example, a Poisson distribution is assumed for modeling technical variations in popular tools for identifying differentially expressed genes (such as edgeR^4^ and DESeq^5^) and in statistical methods for clustering genes^6^ or samples^7^. However, this assumption is primarily based on the argument that sequencing data represent discrete counts, and the supporting empirical evidence is very limited^8^. In addition, this empirical evidence was derived from technical replicates for the read generation step only (i.e., two aliquots of the same library allocated to two lanes on a flow cell), and not for the library preparation step.

We investigated the intrinsic stochasticity for the sequencing of microRNAs (miRNAs; a class of small non-coding RNAs) on the basis of data from technical replicates encompassing both the library preparation step and the read generation step. We collected miRNA sequencing data for two sarcomas: a myxofibrosarcoma (MXF) and a pleomorphic malignant fibrous histiocytoma (PMFH), each subjected to library preparation and sequencing six times using uniform experimental handling. We observed that the stochasticity for miRNA sequencing data is more consistent with a gamma distribution and provided a biological interpretation based on the exponential stochastic growth of PCR amplifications. We further validated this observation in two independent datasets, one for miRNA sequencing and another for RNA sequencing. Motivated by the gamma distributed stochasticity, we provided a simple and powerful method (based on cubic root transformation and normal-distribution based methods) for analyzing RNA sequencing data and showed its superiority to three existing methods for differential expression analysis using three data examples of technical replicate data and biological replicate data.

## Results

### Empirical data indicate a gamma distribution for the stochasticity assumption of RNA-seq data

**Figure 1:**
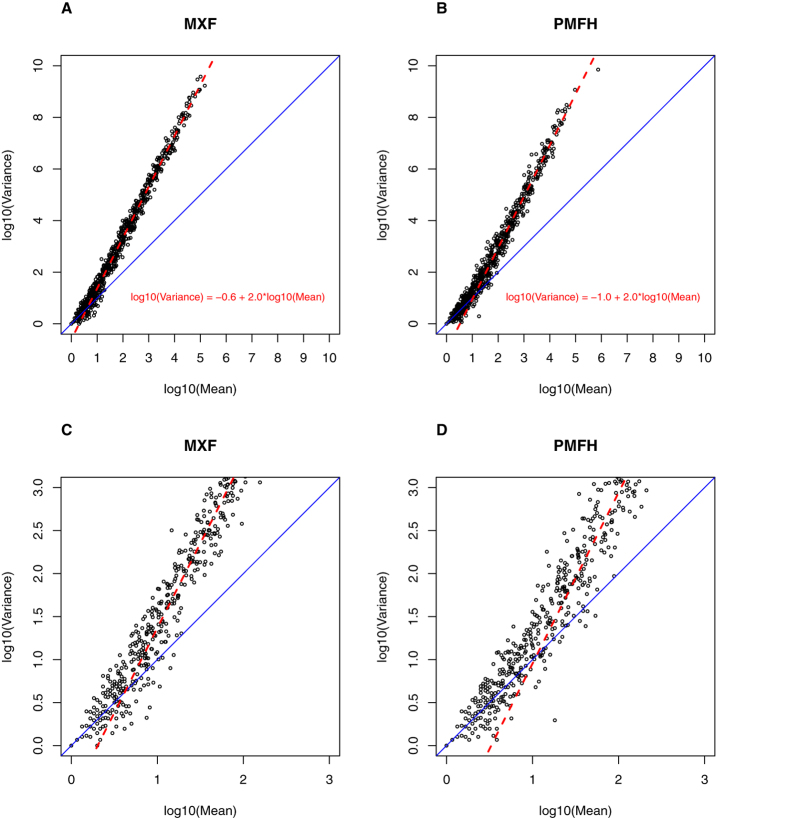
Scatter plots of miRNA-specific variance versus the miRNA-specific mean number of reads on the logarithmic scale for the MXF sample (A) and the PMFH sample (B). Panels (**C,D**) focus on the low-read portion of the same plots. Blue solid line is the diagonal. Red dashed line is the fitted straight line for the high-read miRNAs in each sample, with the formula of the fitted line provided in red.

**Figure 2:**
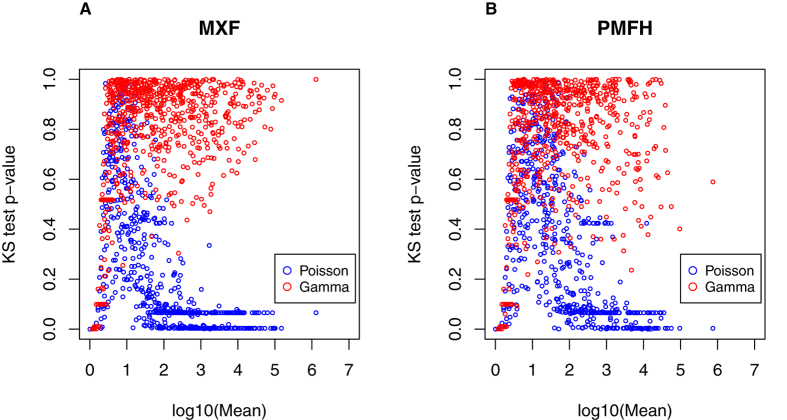
Scatter plots of the miRNA-specific p-values for the Kolmogorov-Smirnov goodness-of-fit test assuming a Poisson distribution (blue points) or a gamma distribution (red points) versus the miRNA-specific logarithmic mean. (**A**) MXF; (**B**) PMFH.

Supplementary Figures S1 and S2 show the overall distribution of the sarcoma sextuplicate data. For each miRNA in each sample, we calculated the mean and variance of the sequencing reads across the six technical replicates. There was a distinct mean-variance relationship that was dependent on the mean (Fig. 1). For low-read miRNAs (roughly, mean reads <10 for MXF and <30 for PMFH), variance approximately equaled the mean; for the remaining miRNAs (defined as high-read miRNAs), variance scaled approximately with the square of the mean. The former mean-variance relationship is consistent with a Poisson distribution, possibly due to the rarity of the low-read miRNAs; however, the latter is consistent with a gamma distribution^9^. To further assess this inference, for each miRNA we calculated the p-value for the Kolmogorov–Smirnov goodness-of-fit test assuming either a Poisson distribution or a gamma distribution, and plotted each of them against the mean (Fig. 2). This analysis confirmed that miRNAs with low reads tend to follow a Poisson distribution (that is, p-values uniformly distributed across the p-value scale between 0 and 1), but miRNAs with high reads are more consistent with a gamma distribution.

In addition to our own data, we also observed the same mean-variance relationship in miRNA sequencing data from the miRNA Quality Control Study^10^ (Supplementary Figure S3) and in RNA sequencing data from the Sequencing Quality Control Consortium^3^ (Supplementary Figure S4). These findings support the robustness of our results and their potential generalizability to RNA sequencing.

**Figure 3:**
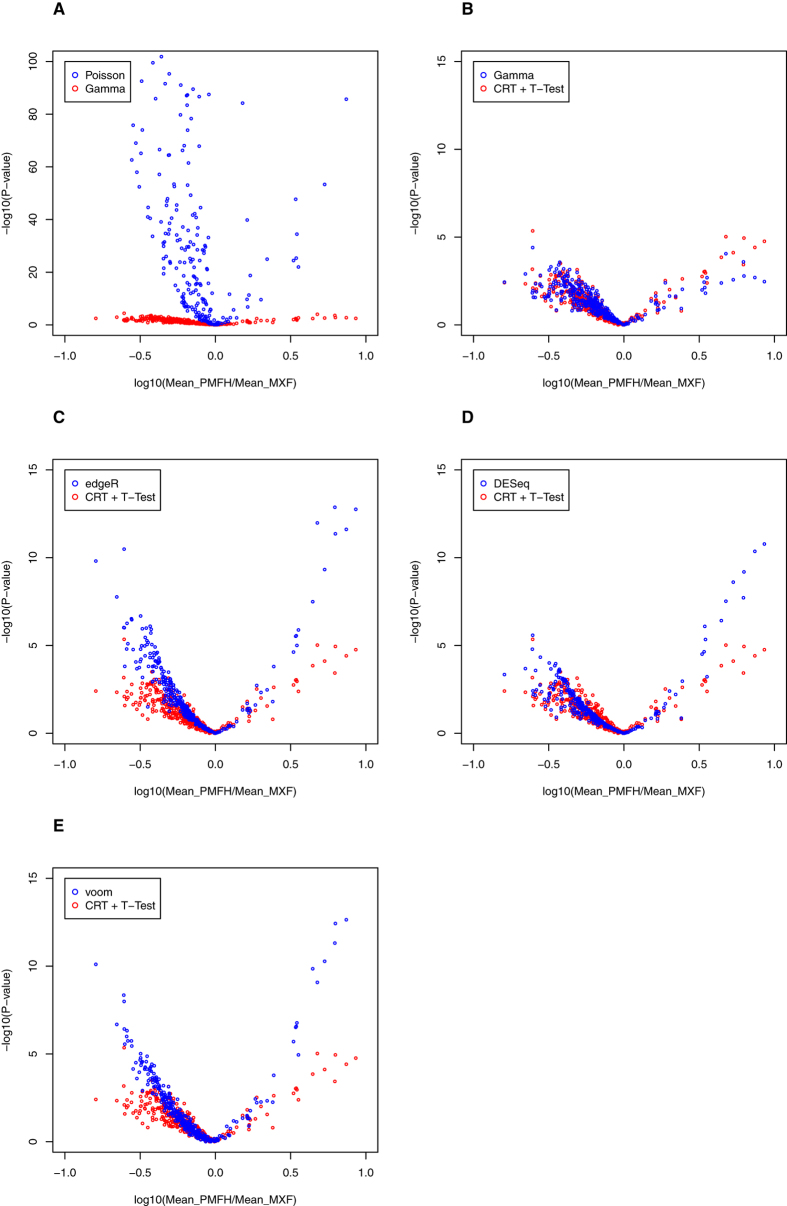
(**A**) Volcano plot of fold change and statistical significance for differential miRNA expression. The miRNA-specific –log10(p-value) for comparing MXF and PMFH based on a Poisson distribution assumption (blue points) or a gamma distribution assumption (red points) is plotted against the miRNA-specific logarithmic mean ratio between MXF and PMFH. (**B–E**) Volcano plots comparing the p-values for differential miRNA expression based on the two-sample t-test after cubic root transformation (CRT) (red points) versus the p-values based on the generalized linear model method assuming a gamma distribution (blue points) (**B**), edgeR (blue points) (**C**), DESeq (blue points) (**D**), and voom (blue points) (**E**).

To demonstrate the importance of the stochasticity assumption in the analysis of sequencing data, we investigated its effect on the analysis of differential expression when only technical replicates are available (e.g., in the analysis of pooled samples). Because such experiments typically involve only a small number of technical replicates, the variance is typically approximated as a function of the mean under the constraint imposed by a distribution, rather than being estimated empirically, to avoid the statistically undesirable consequences of estimating both mean and variance from sparse data. Our analysis compared the mean expression level between MXF and PMFH for each of their 352 shared high-read miRNAs (Supplementary Table S1) and approximated the variance under the gamma distribution or the Poisson distribution. Both the gamma-based test and the Poisson-based test were performed under the generalized linear model framework^11^. Figure 3A compares the p-values resulted from the two tests. The Poisson-based test resulted in much smaller p-values, due to severe underestimation of the variance when imposing the assumption that variance equals the mean, and hence led to many false-positive discoveries. Fourteen miRNAs had a Poisson-based p-value less than 0.0001 and a gamma-based p-value greater than 0.5. The boxplots for these 14 miRNAs (Supplementary Figure S5) indicate a clear lack of significant differences in their mean abundance between MXF and PMFH.

### The gamma distribution can be explained by the exponential stochastic growth of PCR amplification

The gamma-distributed nature of RNA-seq data can be explained by the use of PCR amplification in library preparation and in the initial step of read generation. PCR amplifies a molecule exponentially: with each cycle, a molecule is doubled with a certain probability (i.e., the amplification efficiency)^2^. Over multiple cycles, each molecule in the starting sample can evolve in many different directions depending on whether it is doubled at each cycle. The probability distribution of the amplified count for each molecule hence fits an exponential distribution. When a miRNA is represented by multiple molecules in the initial tissue sample, its count after PCR amplifications is the sum of the amplified count of each molecule; therefore, its distribution is the sum of exponential distributions, which is represented by a gamma distribution^12^. In short, sequencing reads count the exponentially amplified product of the miRNA molecules, whose stochasticity is better approximated by a gamma distribution than a Poisson distribution. By this reasoning, the gamma distribution is likely to be relevant to the stochasticity of any quantification with methods involving PCR.

### Gamma distribution implies a simple and powerful method based on cubic root transformation for the analysis of RNA-seq data

Although our method of analyzing the gamma distribution under the generalized linear model requires statistical expertise, gamma distributions can be analyzed simply and accurately by the use of cubic root transformation and normal-distribution–based methods, such as the t-test for two-group comparison^13^,^14^. We applied this simple method to compare the two sets of sextuplicates, and found an excellent agreement with the more sophisticated method based on the generalized linear model (Fig. 3B). This immediately applicable solution is readily accessible to both quantitative and non-quantitative scientists, for studies with technical replicates (such as studies of cell lines or pooled samples^15^,^16^).

Statistical methods such as edgeR^4^, DESeq^5^, and voom^17^ were developed for the analysis of biological replicates, allowing for the variance to exceed the mean. They could in principle be used to analyze technical replicates as well. We applied these three methods to the sextuplicate data and compared with our method based on cubic root transformation and t-test. For most miRNAs, our method resulted in similar p-values to voom, DESeq, and edgeR (in the order of similarity) (Fig. 3C–E and Supplementary Figure S6). For the miRNAs whose differential expression status differed between methods, our method was more aligned with the empirical evidence (Supplementary Figures S7 and S8).

**Figure 4:**
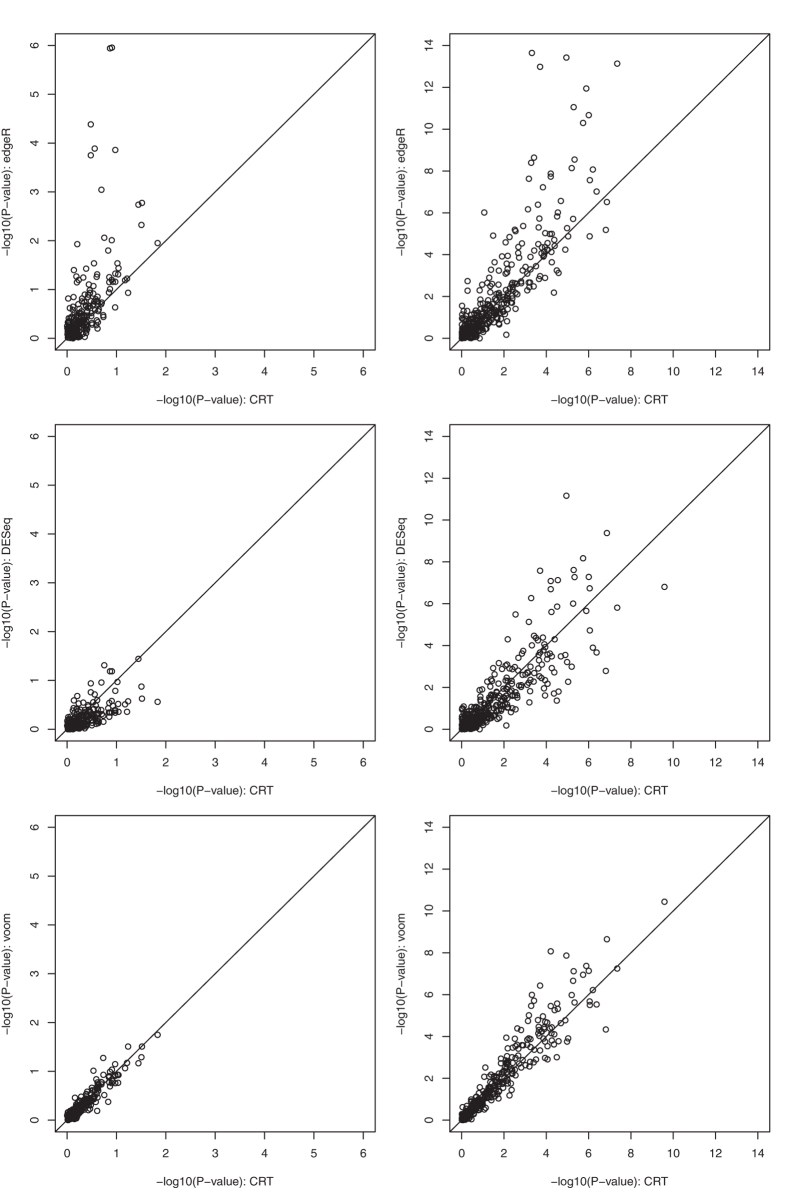
Scatterplot of the −log10(p-value) for differential miRNA expression based on the two-sample t-test after cubic root transformation (CRT) versus the −log10(p-value) based on edgeR (top panels), DESeq (middle panels), and voom (bottom panels). The left column shows data for the TCGA ovarian cancer study comparing platinum-sensitive versus platinum-resistant tumors; the right column shows data for a breast cancer study comparing invasive ductal carcinoma versus normal breast tissue. Analysis was done for high-read genes (defined as mean reads >10) for each study.

We hypothesized that cubic root transformation can also help normalize biological replicate data and subsequently allow the use of normal-distribution-based methods such as the two-sample t-test for differential expression analysis. We examined this hypothesis in two public miRNA-seq datasets with biological replicates, one from The Cancer Genome Atlas (TCGA) ovarian cancer study^18^ and another from a breast cancer study^19^, in comparison with edgeR, DESeq, and voom. The analysis, again, showed that (1) our method correlated highly with voom, DESeq, and edgeR (in the order of similarity) (Fig. 4), and (2)) for the miRNAs whose differential expression status differed between methods, our method was more aligned with the empirical evidence (Supplementary Figure S9–S12).

Taken together, for the analysis of both technical replicates and biological replicates, our method provides a good balance between the over-liberal tendency of edgeR and the over-conservativeness of DESeq^20^. In addition, our method is conceptually and computationally much simpler than voom. Moreover, our method allows a body of existing statistical methodologies developed for microarray data to be extended to sequencing data.

## Discussion

In summary, we have made three contributions towards the understanding of fundamental stochastic properties of RNA sequencing: (1) we have provided empirical evidence that the stochastic distribution of RNA sequencing is gamma for high-read genes and Poisson for low-read genes, (2) we have offered a biological interpretation for the gamma distribution by recognizing its connection with exponential PCR amplifications, and (3) we have proposed a simple, powerful, and versatile solution using cubic root transformation and normal-distribution-based methods for the analysis of sequencing data, which is readily assessable to biomedical researchers.

Our finding of the gamma-distributed nature of intrinsic stochasticity has broad implications not only for the assessment of differential expression, but also broadly for applications of deep RNA sequencing and other quantitative methods that involve PCR amplification. In addition, our findings highlight the importance of studying the stochasticity of a technology using technical replications for the entire experiment.

## Methods

### Tumor tissue sample collection

Two pooled soft tissue sarcoma (STS) samples were used in our study. One was from pooling 27 primary myxofibrosarcoma (MXF) tumor tissue samples and another from pooling 27 primary pleomorphic malignant fibrous histiocytoma (PMFH) tumor tissue samples. These tumor samples, all from newly diagnosed, previously untreated tumors, were collected at Memorial Sloan Kettering Cancer Center (MSKCC) between 2000 and 2012. Detailed description of these 54 tumors will be provided in a separate manuscript that is under preparation. Human tumor tissues used in this study were obtained from participants who provided informed consent in written form and their use in our study was approved by the MSKCC Institutional Review Board. The methods in our study were carried out in accordance with the approved guidelines.

### Tumor microdissection and RNA extraction

Sample preparation used strict quality control on the specimens. Freshly harvested tissue was snap-frozen for eventual cryomold embedding and RNA extraction for sequencing analysis. Cryomolds (0.5 × 1 × 1 cm) were macrodissected under the supervision of a dedicated soft tissue sarcoma pathologist to ensure subtype uniformity and to eliminate necrotic/normal tissue as described^21^. RNA was isolated from approximately thirty 30-mm cryosections corresponding to approximately 20 mg of tissue, using the first and last section to assess tumor content; only samples containing 50% or greater tumor content were further characterized. The tissues were homogenized in TRIzol (Invitrogen) using a Polytron instrument (polytron, PT, MR2100; Kinematica AG) for 1 minute, and total RNA was isolated by a modified TRIzol protocol^19^. Total RNA yield was assessed using a nanodrop spectrophotometer; quality of isolated RNA was assessed using an Agilent Bioanalyzer and a 1% agarose gel based on the relative abundance of 18 S and 28 S subunits of ribosomal RNA.

### Small RNA sequencing and mapping

Each pooled STS sample was sequenced six times using consistent experimental handling. We used a barcoded small RNA sequencing approach^22^. We mapped the reads using a bioinformatics pipeline as described^23^. Briefly, we selected reads with an insert of 16 to 25 nt. Adapter sequences were extracted from sequence reads using the following criteria: 4-nt minimum overlap of 3′ adapter or 5-nt minimum 3′ overlap of adapter with 1 mismatch excluding insertions and deletions in the first nucleotide of the adapter past the barcode. Barcodes were assigned without allowing any mismatches. The miRNA sequencing count data will be available upon request to the first author.

### Statistical analysis

For each gene in each of the two STS pooled samples, we calculated the mean and variance of the reads count across the six technical replicates. For each sample, we then plotted the mean plus one versus the variance plus one among the genes on the logarithmic scale. To evaluate the evidence of goodness-of-fit for an assumed probability distribution, for each gene in each pooled sample, we performed the Kolmogorov–Smirnov test and calculated the p-value, under the assumption of a Poisson distribution and under the assumption of a gamma distribution^9^. To assess the evidence against the null hypothesis of equivalent expression in MXF and PMFH, we compared the two sets of sextuplicates using the generalized linear model as implemented in R package glm2. More specifically, we used the glm function with the covariate being an indicator function for sample group (MXF versus PMFH) and the identity link; we used the Poisson family for the Poisson distribution assumption and the gamma family for the gamma distribution assumption. In addition, we compared the two sets of sextuplicates using cubic root transformation followed by a two-sample t-test^13^,^14^. For the purpose of comparison, we also compared the two sets of sectuplicates using edgeR^4^, DESeq^5^, and voom^17^. Both edgeR and DESeq assume negative binomial as the marginal distribution, while voom applies logarithm transformation to the count data and then uses normal-based methods with weights derived from mean-variance-relationship of the transformed data.

Scatterplots of miRNA-specific variance versus the miRNA-specific mean number of reads were drawn for data from the miRQC study^10^ (GSE49816) and data from the SEQC study^3^ (GSE49712). For the former study, we calculated the mean and variance of the reads count across the two technical replicates for sample A combined with two replicates of sample C and across the two replicates of sample B combined with two replicates of sample D. For the latter study, we calculated the mean and variance of the reads count across the four replicates of sample A and across the four replicates of sample B.

We performed differential miRNA expression, using cubic root transformation followed by t-test, edgeR, DESeq, and voom, using data from the TCGA ovarian cancer study^18^ comparing platinum-sensitive versus platinum-resistant tumors and data from a breast cancer study^19^ comparing invasive ductal carcinoma versus normal breast tissue. For the former study, platinum status data was from the original publication’s supplementary materials and sequencing data (reads per million) from the TCGA data portal. For the latter study, tissue type data and sequencing data (reads per million) were both from the original publication’s supplementary materials. Analysis was done for high-read genes (defined as mean reads >10) for each study.

Statistical analyses were conducted using R^24^.

## Additional Information

**How to cite this article**: Qin, L.-X. *et al*. Empirical insights into the stochasticity of small RNA sequencing. *Sci. Rep*. **6**, 24061; doi: 10.1038/srep24061 (2016).

## Supplementary Material

Supplementary Information
