# Supplementary material for: Empirical insights into the stochasticity of small RNA sequencing
Source: Sci Rep. 2016 Apr 7;6:24061. doi: 10.1038/srep24061 (PMC4823707; doi:10.1038/srep24061)
Supplement: Supplementary Information [file srep24061-s1.pdf]

Empirical insights into the stochasticity of small RNA sequencing

Li-Xuan Qin[1]*, Thomas Tuschl[2#], Samuel Singer[3#]

[1]Department of Epidemiology and Biostatistics, Memorial Sloan Kettering Cancer Center, New York, NY

[2]Laboratory of RNA Molecular Biology, The Rockefeller University, New York, NY

[3]Department of Surgery, Memorial Sloan Kettering Cancer Center, New York, NY

[#]Equal Contribution

***Corresponding Author**

Li-Xuan Qin

email: qinl@mskcc.org

phone: 646-888-8251

Department of Epidemiology and Biostatistics

Memorial Sloan Kettering Cancer Center

1275 York Ave

New York, NY10065

SUPPLEMENTARY FIGURE LEGENDS

Figure S1. Boxplot of the two sets of sextuplicates. Each box represents a replicate. Replicates for MXF are plotted with unfilled boxes, and replicates for PMFH are plotted in grey boxes. The y-axis is the number of reads (plus one to account for zero-read miRNAs) on the logarithmic scale.

Figure S2. Scatterplot between replicates for each sextuplicate: A. MXF; B, PMFH.

Figure S3. Scatterplots of miRNA-specific variance versus the miRNA-specific mean number of reads for data from the miRQC study (GSE49816). The data shown are two replicates of sample A combined with two replicates of sample C (panel A) and two replicates of sample B combined with two replicates of sample D (panel B), both plotted on the logarithmic scale. Blue solid line is the diagonal. Red dashed line is the fitted straight line for the high-read miRNAs (defined as mean reads >10) in each sample, with the formula of the fitted line provided in red.

Figure S4. Scatterplots of gene-specific variance versus the gene-specific mean number of reads for data from the SEQC study (GSE49712). The data shown are for four replicates of sample A (panel A) and four replicates of sample B (panel B), plotted on the logarithmic scale. Blue solid line is the diagonal. Red dashed line is the fitted straight line for the high-read genes (defined as mean reads >10 for sample A and >100 for sample B) in each sample, with the formula of the fitted line provided in red.

Figure S5. Boxplots by sample type for the 14 miRNAs that have a Poisson-based p-value less than 0.0001 and a gamma-based p-value greater than 0.5.

Figure S6. Scatterplot of the p-values for differential miRNA expression based on the two-sample t-test after cubic root transformation (CRT) versus the p-values based on the generalized linear model (GLM) method, edgeR, DESeq, and voom, comparing MXF versus PMFH in the sarcoma study.

Figure S7. Boxplots by sample type for the miRNAs that have an edgeR-based p-value less than 0.01 and a CRT-based p-value greater than 0.1, comparing MXF versus PMFH in the sarcoma study.

Figure S8. Boxplots by sample type for the miRNAs that have a voom-based p-value less than 0.01 and a CRT-based p-value greater than 0.1, comparing MXF versus PMFH in the sarcoma study.

Figure S9. Boxplots by sample type for the miRNAs that have an edgeR-based p-value less than 0.01 and a CRT-based p-value greater than 0.1, comparing platinum-sensitive versus platinum-resistant patients in the TCGA ovarian cancer study.

Figure S10. Boxplots by sample type for the miRNAs that have an edgeR-based p-value less than 0.01 and a CRT-based p-value greater than 0.1, comparing invasive ductal carcinoma versus normal breast tissue in the Farazi et al. (2011) study.

Figure S11. Boxplots by sample type for the miRNAs that have a CRT-based p-value less than 0.01 and a edgeR -based p-value greater than 0.1, comparing invasive ductal carcinoma versus normal breast tissue in the Farazi et al. (2011) study.

Figure S12. Boxplots by sample type for the miRNAs that have a CRT-based p-value less than 0.01 and a DESeq-based p-value greater than 0.1, comparing invasive ductal carcinoma versus normal breast tissue in the Farazi et al. (2011) study.

Supplementary Figure S1.

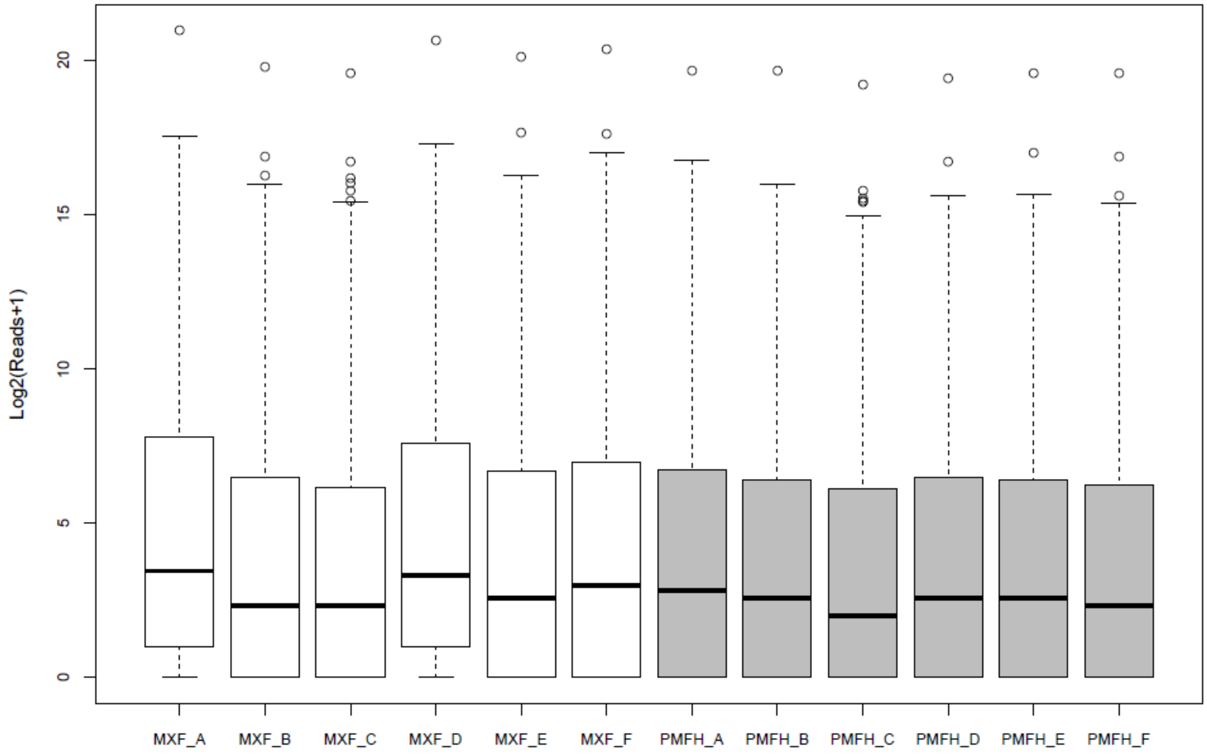

Supplementary Figure S2.

A

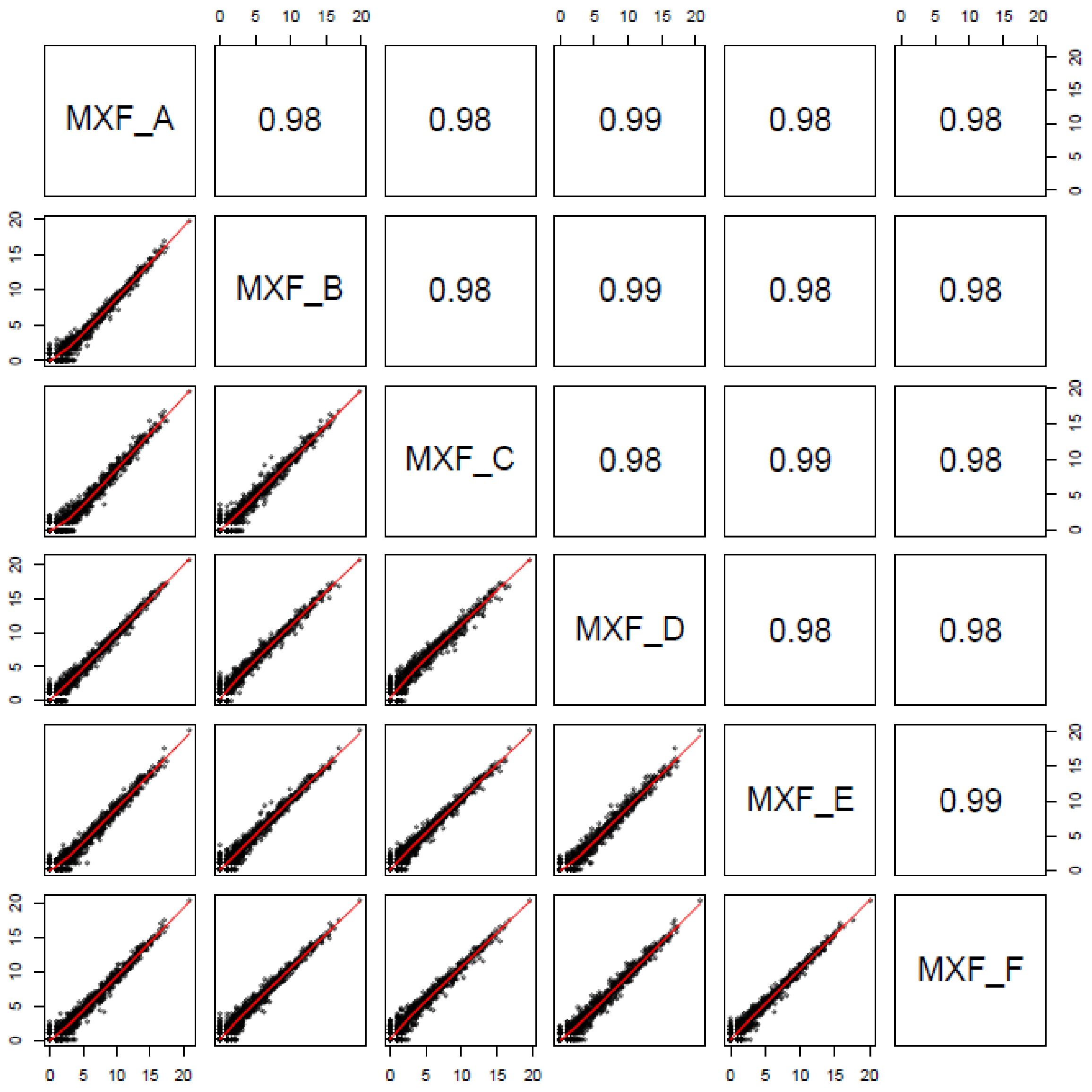

B

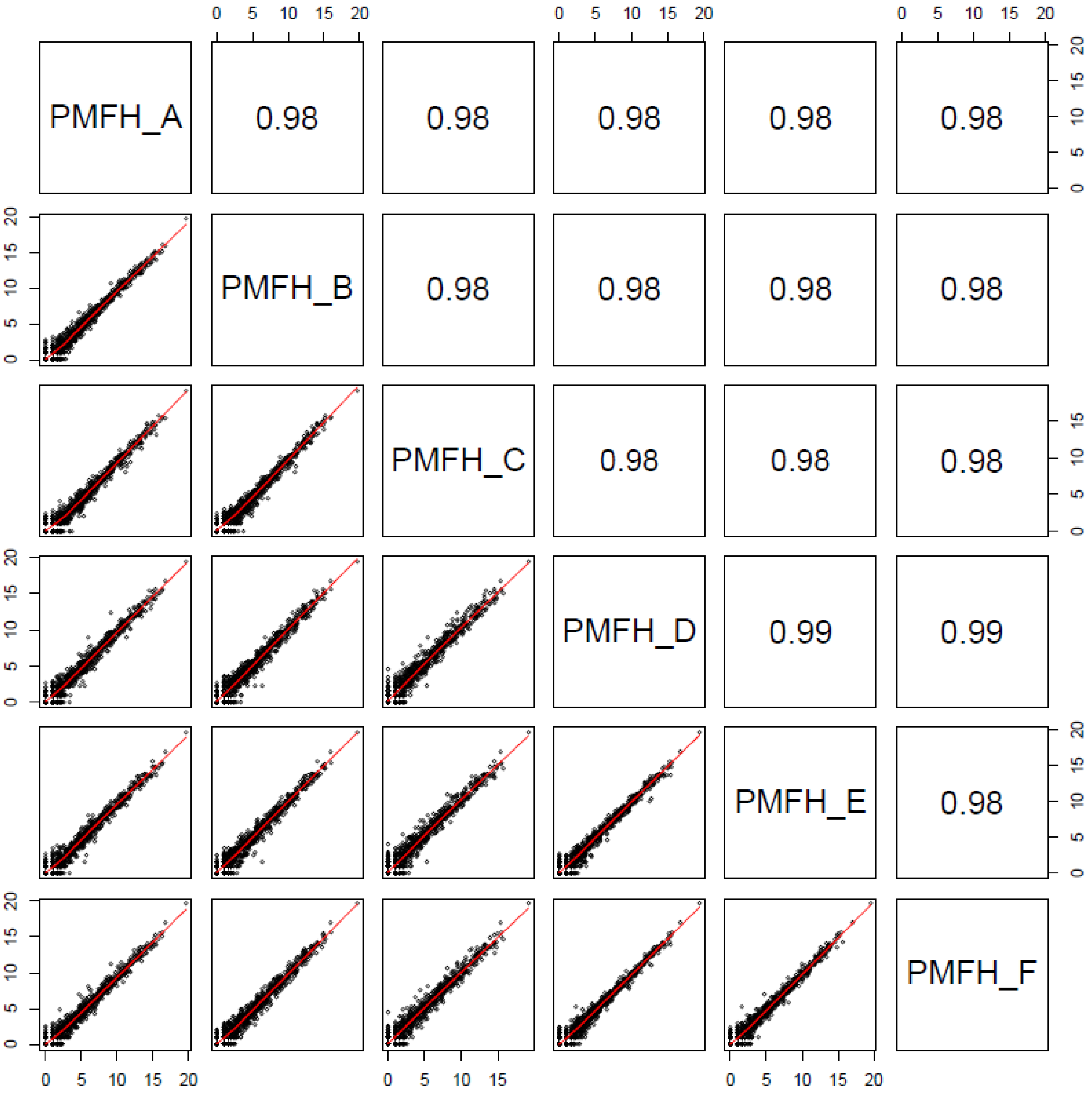

Supplementary Figure S3.

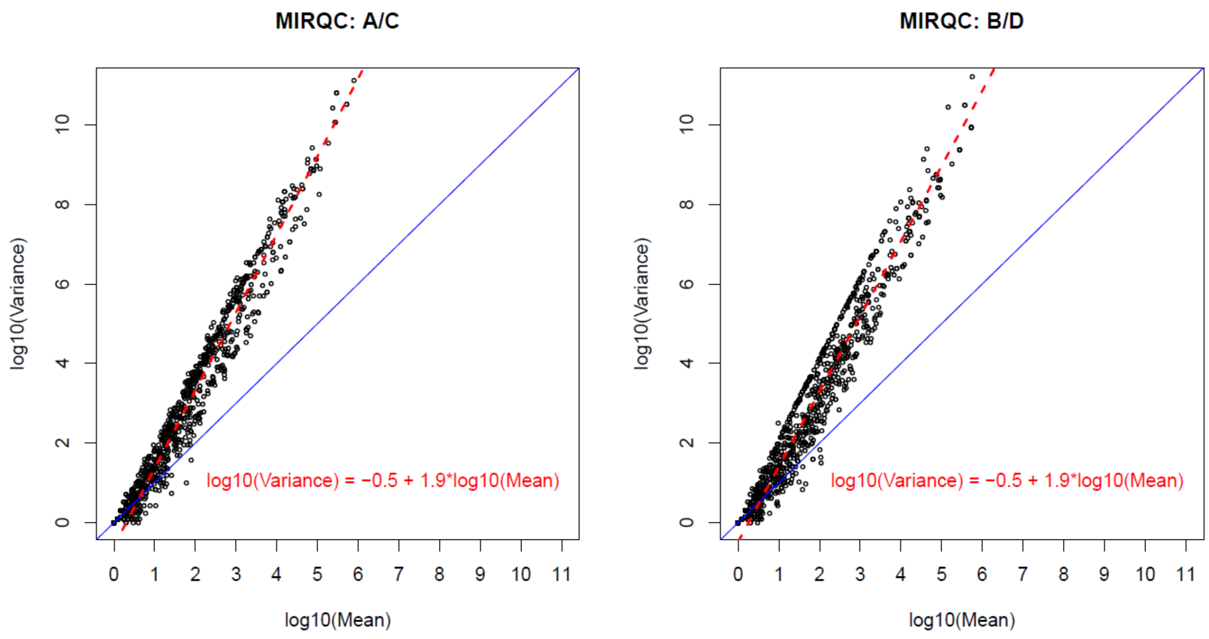

Supplementary Figure S4.

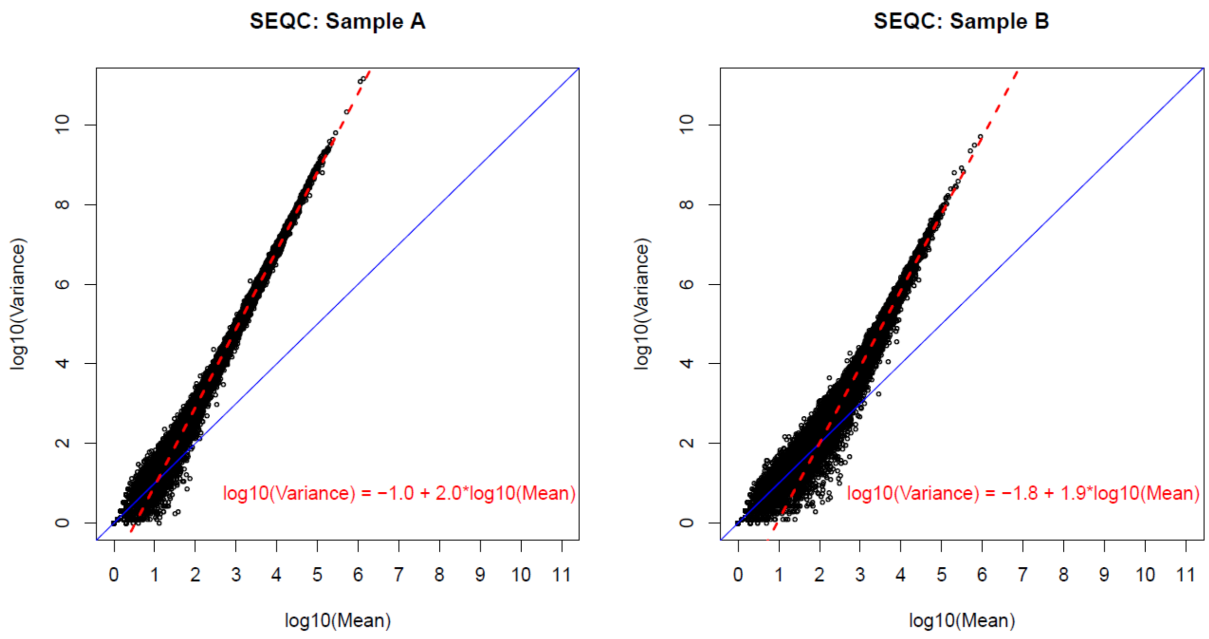

Supplementary Figure S5.

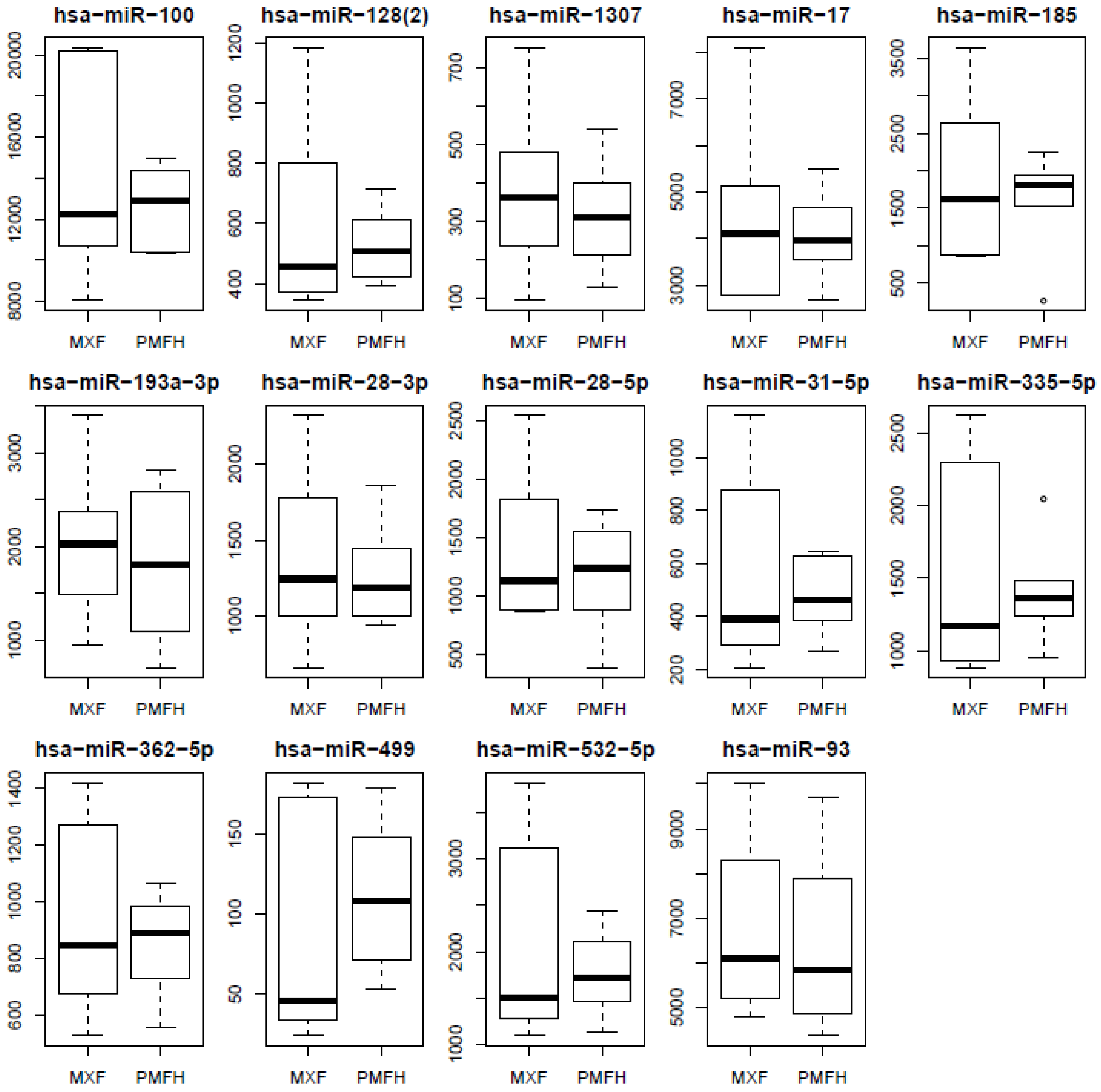

Supplementary Figure S6.

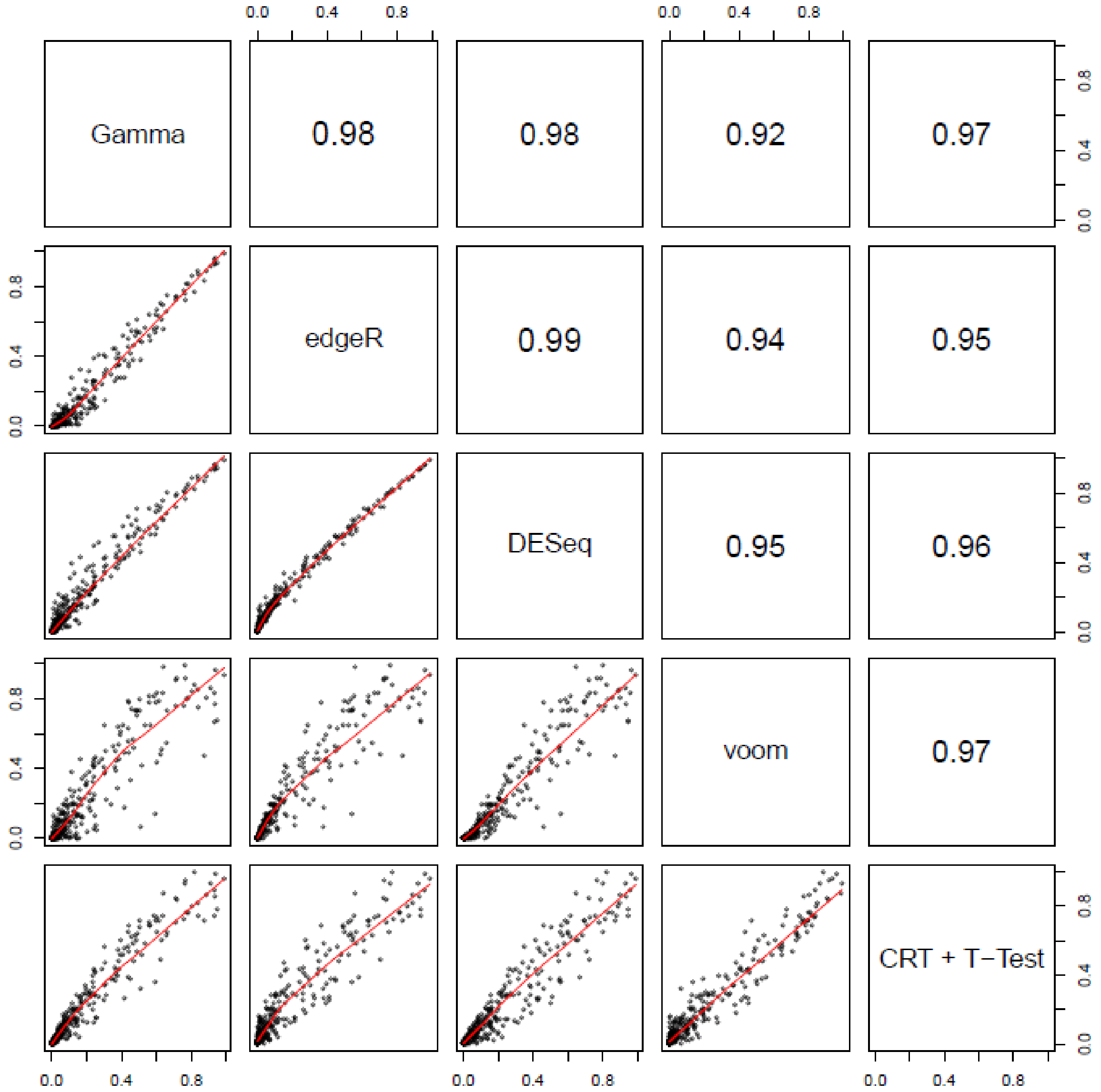

Supplementary Figure S7.

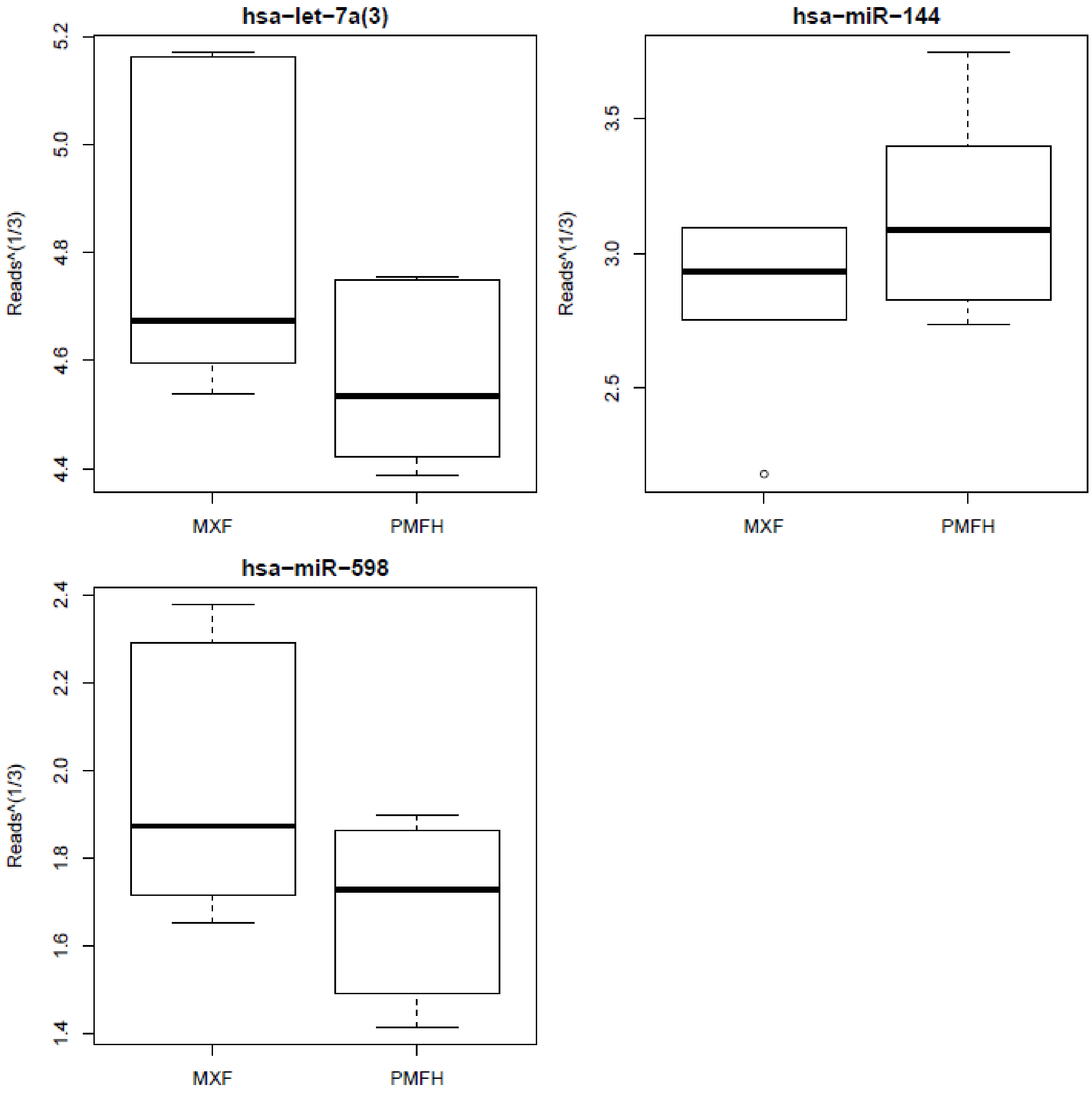

Supplementary Figure S8.

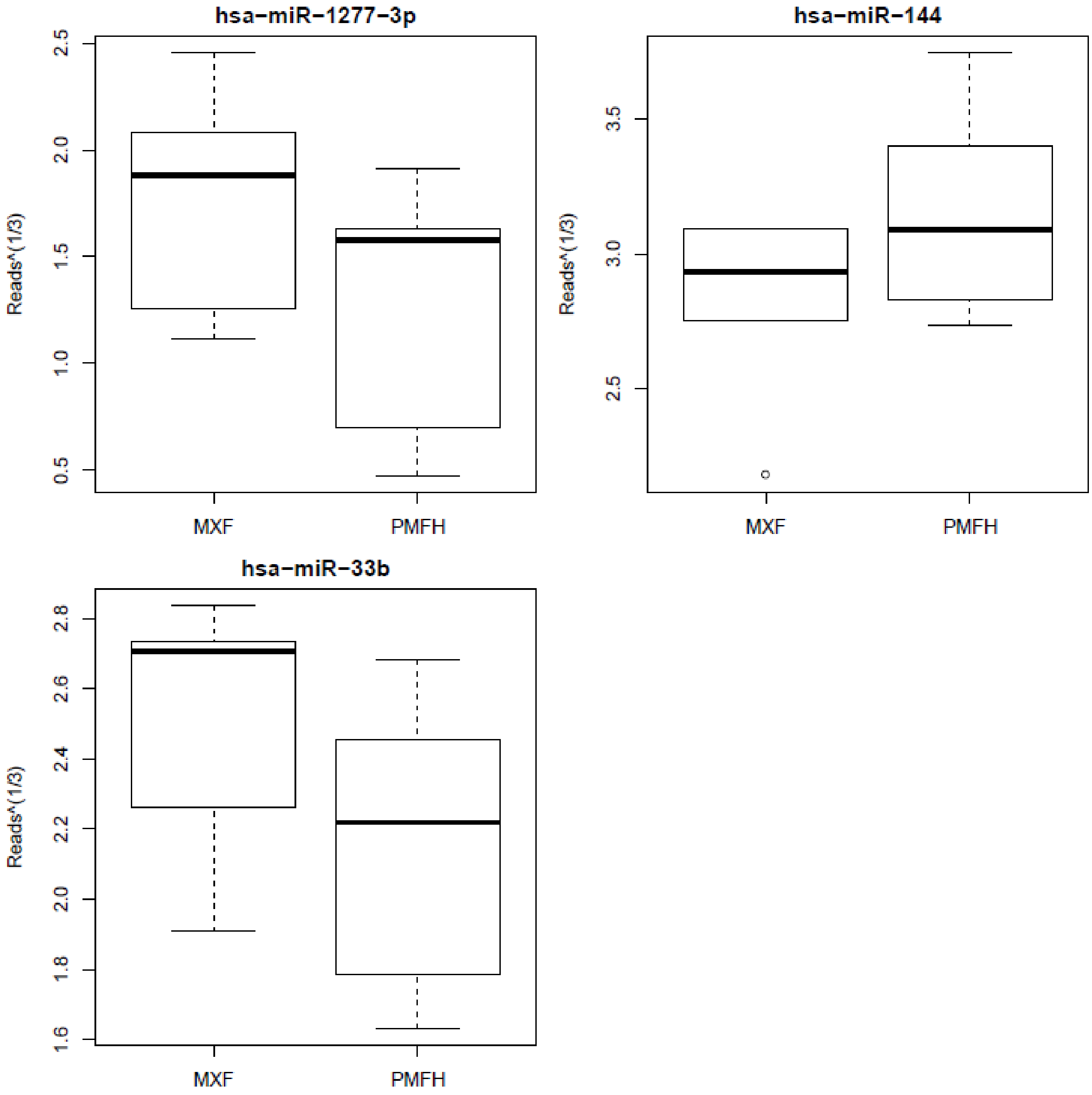

Supplementary Figure S9.

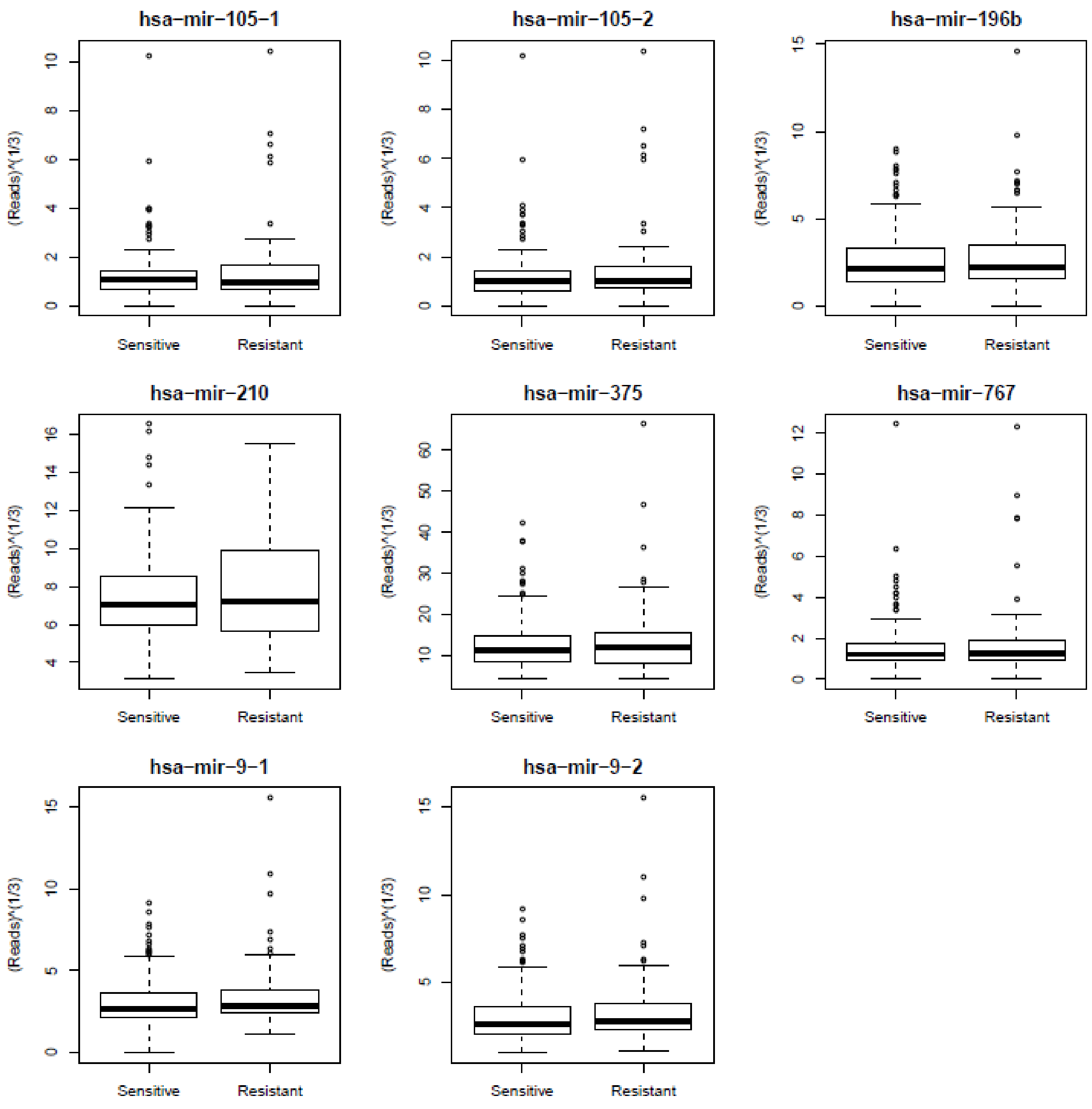

Supplementary Figure S10.

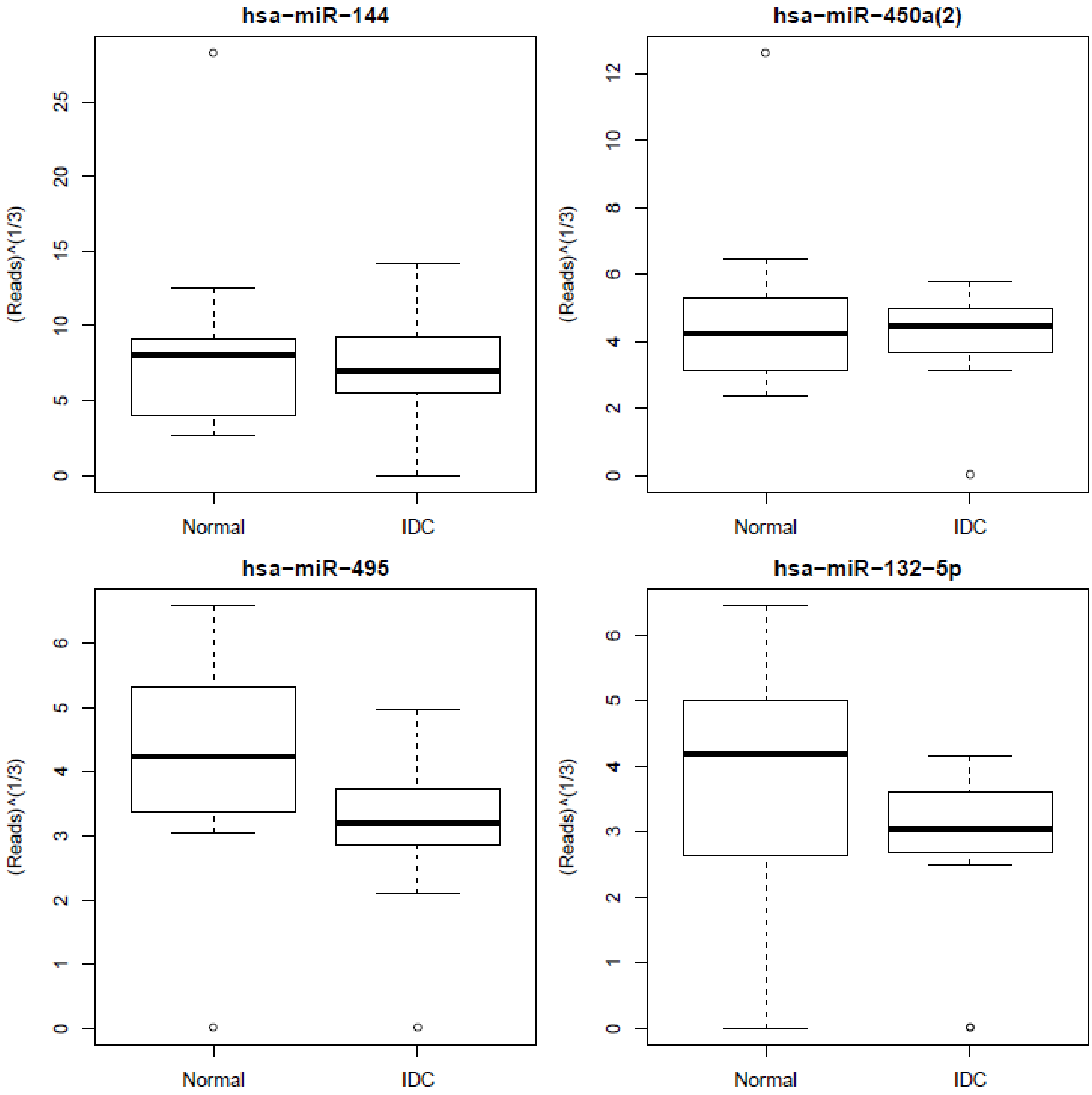

Supplementary Figure S11.

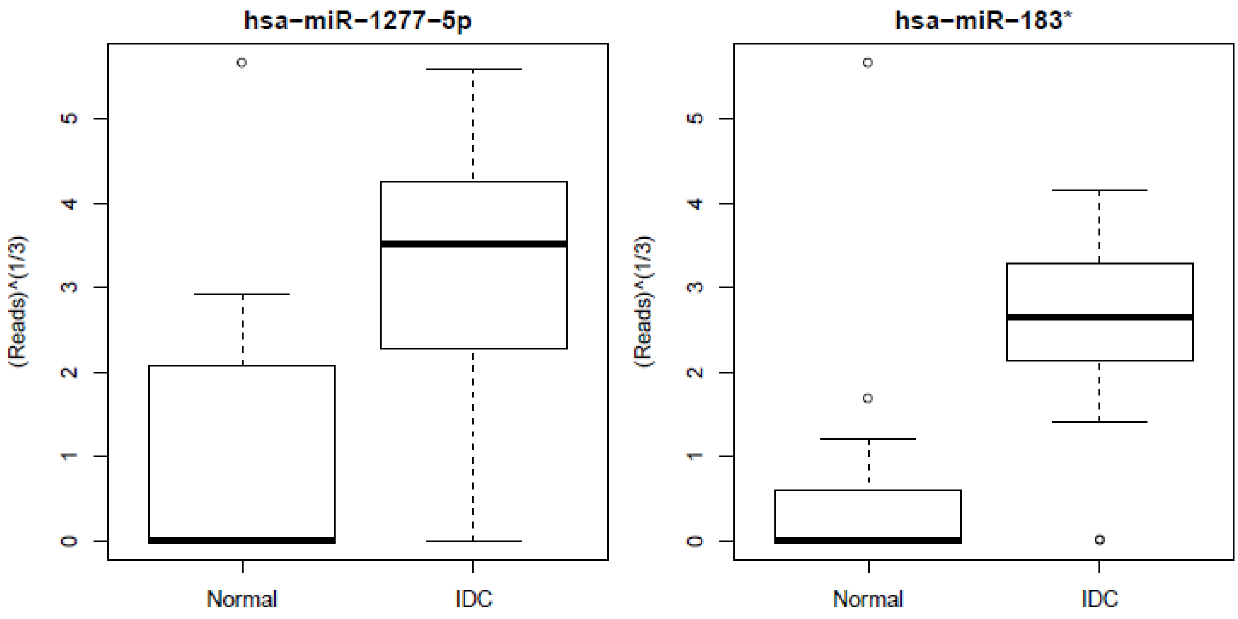

Supplementary Figure S12.

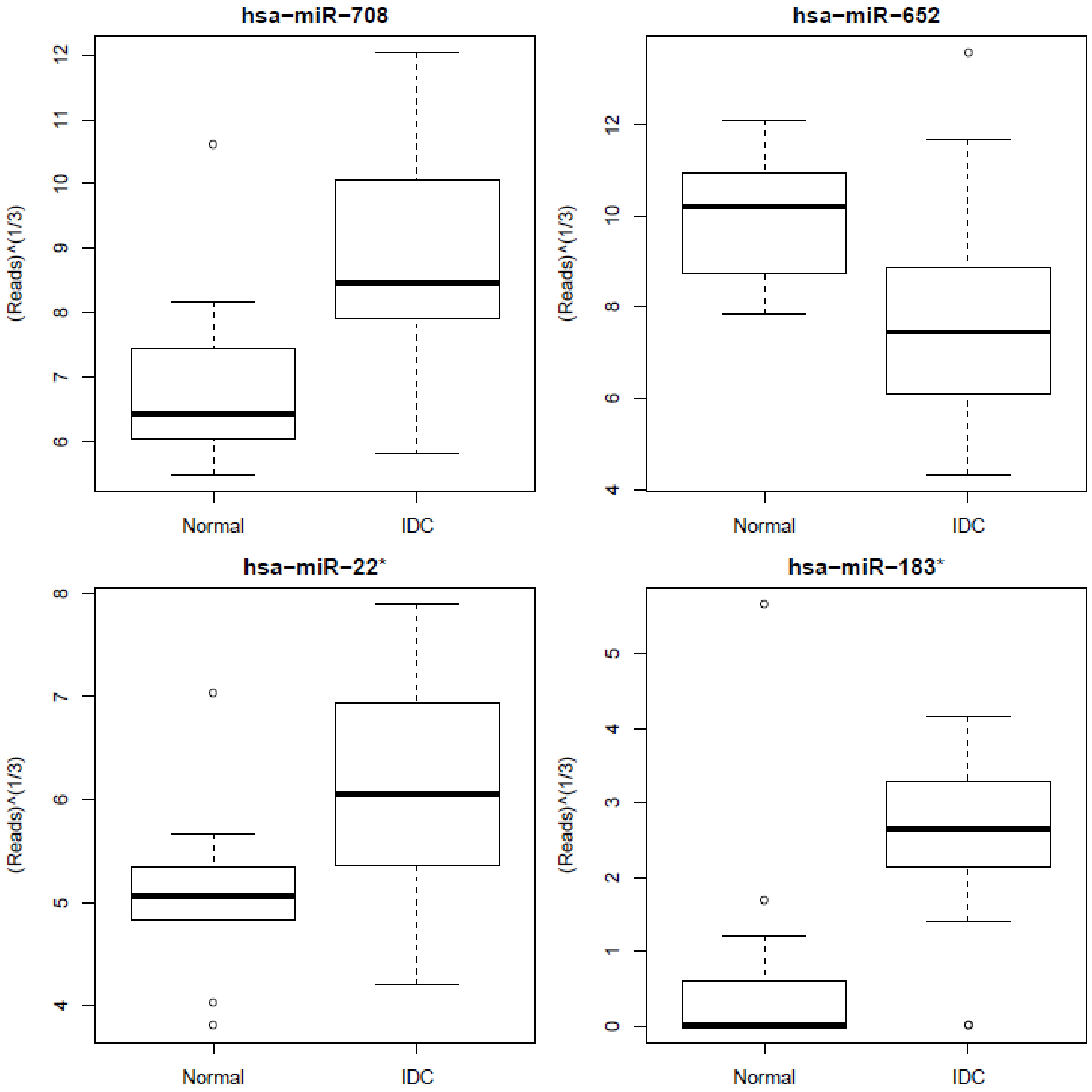

Table S1. Numbers of miRNA reads categories by abundance groups.

	MXF	PMFH	Shared
high-read[a]	485	352	352
low-read	430	553	368
0 reads in all replicates	118	128	66

[a]Mean reads ≥10 for MXF and ≥30 for PMFH
